# Health system factors that influence diagnostic and treatment intervals in women with breast cancer in sub-Saharan Africa: a systematic review

**DOI:** 10.1186/s12889-021-11296-5

**Published:** 2021-07-06

**Authors:** Gloria Gbenonsi, Mouna Boucham, Zakaria Belrhiti, Chakib Nejjari, Inge Huybrechts, Mohamed Khalis

**Affiliations:** 1 International School of Public Health, Mohammed VI University of Health Sciences, Casablanca, Morocco; 2 National School of Public Health, Rabat, Morocco; 3 International Agency for Research on Cancer, Lyon, France

**Keywords:** Breast cancer, Health system, Diagnostic interval, Treatment interval, Sub-Saharan Africa

## Abstract

**Background:**

Breast cancer patients in sub-Saharan Africa experience long time intervals between their first presentation to a health care facility and the start of cancer treatment. The role of the health system in the increasing treatment time intervals has not been widely investigated. This review aimed to identify existing information on health system factors that influence diagnostic and treatment intervals in women with breast cancer in sub-Saharan Africa to contribute to the reorientation of health policies in the region.

**Methods:**

PubMed, ScienceDirect, African Journals Online, Mendeley, ResearchGate and Google Scholar were searched to identify relevant studies published between 2010 and July 2020. We performed a qualitative synthesis in accordance with the Preferred Reporting Items for Systematic Reviews and Meta-Analyses (PRISMA) statement. Related health system factors were extracted and classified according to the World Health Organization’s six health system building blocks. The quality of qualitative and quantitative studies was assessed by using the Critical Appraisal Skills Program Quality-Assessment Tool and the National Institute of Health Quality Assessment Tool, respectively. In addition, we used the Confidence in the Evidence from Reviews of Qualitative Research tool to assess the evidence for each qualitative finding.

**Results:**

From 14,184 identified studies, this systematic review included 28 articles. We identified a total of 36 barriers and 8 facilitators that may influence diagnostic and treatment intervals in women with breast cancer. The principal health system factors identified were mainly related to human resources and service delivery, particularly difficulty accessing health care, diagnostic errors, poor management, and treatment cost.

**Conclusion:**

The present review shows that diagnostic and treatment intervals among women with breast cancer in sub-Saharan Africa are influenced by many related health system factors. Policy makers in sub-Saharan Africa need to tackle the financial accessibility to breast cancer treatment by adequate universal health coverage policies and reinforce the clinical competencies for health workers to ensure timely diagnosis and appropriate care for women with breast cancer in this region.

**Supplementary Information:**

The online version contains supplementary material available at 10.1186/s12889-021-11296-5.

## Background

Breast cancer is the most common cancer among women worldwide, with an estimated 6,875,099 five-year prevalent cases between 2013 and 2018 [1]. Breast cancer accounted for 24.2% of all new cancer cases among females in 2018 [1]. Despite advances in cancer therapy, there are significant differences in survival rates between developing and developed countries [2, 3]. More than 67% of breast cancer deaths worldwide occur in low-and middle-income countries (LMICs), including sub-Saharan Africa [1]. With 55,938 estimated deaths in 2018, breast cancer is one of the leading causes of cancer death among women in sub-Saharan Africa after cervical cancer [4].

Breast cancer can be preventable and curable with early and adequate screening and management, but it is a fact that, in most developing countries, women with breast cancer are diagnosed very late, mainly due to a lack of information on early detection and insufficient access to health services [5–7].

By 2019, more than 90% of high-income countries reported that their public health systems had comprehensive cancer treatment services, while less than 15% of low-income countries reported comprehensive cancer treatment services [8]. Many studies from LMICs have reported an association between an advanced clinical stage of breast cancer at treatment onset and time intervals of more than three months between symptom discovery and treatment start [9]. The evidence shows that the median of this interval is 30–48 days in high-income countries but 3–8 months in LMICs [9]**.**

In sub-Saharan Africa, delays in diagnosis and treatment and barriers to care experienced by breast cancer patients have been previously explored [6, 10], and many findings show that patients experienced long time intervals between initial symptoms and presentation to a health care facility (patient interval) and between first presentation and definitive diagnosis or treatment (provider interval) [6, 10].

Most studies conducted among sub-Saharan African women were focused mainly on patient interval factors (sociodemographic, cultural and economic related factors), early detection [11, 12], and knowledge, attitudes, and practices of women regarding breast cancer and self-examination [13, 14].

In addition, most of the review studies conducted in the sub-Saharan Africa region were focused on time to presentation, diagnosis and related factors and stages of diagnosis [11, 12]. However, the role of health system-related factors delaying diagnosis and treatment of breast cancer (from presentation to first treatment) has not been well investigated. To the best of our knowledge, this is the first systematic review to focus on health system factors that may explain delay in diagnosis and treatment in women with breast cancer in sub-Saharan Africa. The results from this study will contribute to the improvement of breast cancer health policies in this region.

## Methods

This systematic review was conducted according to the Preferred Reporting Items for Systematic Reviews and Meta-Analyses (PRISMA) statement by Moher et al. [15].

### Protocol and registration

The review protocol was registered in the database of the international prospective register of systematic reviews “PROSPERO” on April 28, 2020, under the number CRD42020182585 and is available at the following link: https://www.crd.york.ac.uk/prospero/display_record.php?ID=CRD42020182585

### Data sources and search strategy

We developed a comprehensive search strategy and conducted an exhaustive search for studies in different databases: Medline (PubMed), ScienceDirect, African Journals Online, Mendeley, ResearchGate and Google Scholar. To make the search exhaustive and identify additional articles, we looked for other sources and carried out country-by-country (48 sub-Saharan African countries) searches. Reference lists of relevant articles were also hand-searched.

The following keywords were combined by Boolean operators “AND”, “OR” and “Not” to obtain several search equations according to the databases:

“Breast cancer”; “Breast carcinoma”; “Breast neoplasm”; “Breast Tumor”; “Factors”; “Determinants”; “Barriers”; “Challenges”; “Facilitators”; “Opportunities”; “Delayed treatment”; “Time-to-Treatment”; “Provider delay”; “Doctor delay”; “Treatment delay”; “Health system delay”; “Healthcare delivery”; “healthcare access”; “health service accessibility”; “Africa”; “sub-Saharan Africa”; and the names of each of the 48 sub-Saharan African countries (details are provided in supplementary data: Table S4).

### Inclusion and exclusion criteria

Articles were eligible for inclusion in this systematic review if they reported findings from primary research studies conducted among women with breast cancer in sub-Saharan Africa, addressed health system factors influencing the time-to-treatment of women with breast cancer, and were published between January 2010 and 2020. There were no language restrictions, and there were no prior restrictions regarding the study design (qualitative, quantitative, or mixed methods). Studies without abstracts or for which the full text was not available were excluded. Additionally, we excluded studies that mixed female and male breast cancer in their results. No studies were excluded after quality assessment.

### Study selection and data collection process

Zotero reference manager software [16] was used to organize and detect duplicate references. We identified eligible articles by using the PRISMA flow diagram. The first and second authors independently screened all titles and abstracts identified by the search, and those clearly irrelevant to the topic were excluded. The full texts of all potentially eligible papers were retrieved and reviewed for inclusion in this review according to the inclusion criteria. All included studies were independently reviewed by two authors to confirm eligibility (GG and MB).

### Data extraction and items

For the included studies, two authors (GG and MB) independently extracted information such as the characteristics of the study (title, authors, year of publication, country, study design, research method, age group, participants, and sample size), health system factors (barriers and facilitators), and the time to treatment (if available).

All health system factors were classified according to the World Health Organization (WHO) Health Systems Framework’s six building blocks [17], namely: 1) health service delivery, 2) health workforce, 3) heath information systems, 4) access to essential medicines and technologies, 5) health system financing, and 6) leadership and governance. Any discrepancies in the process of selection and extraction were resolved through discussion, if necessary, with two other authors (MK and IH).

As reported in the Aarhus statement’s Guidelines [18], we define “diagnostic interval” as the time between first presentation and diagnosis, and “treatment interval” as the time between diagnosis and treatment start.

### Quality assessment

The quality of the qualitative studies was assessed by using the Critical Appraisal Skills Program (CASP) Quality-Assessment Tool (http://www.casp-uk.net) [19]. The quality of the quantitative studies was assessed using the National Institute of Health (NIH) Quality Assessment Tool for Observational Cohort and Cross-Sectional Studies [20]. Study quality was assessed according to the following criteria: research question, study population, eligibility criteria of the population, sample size justification, outcome measures, response and follow-up rates, statistical analyses, and ethical issues. In addition, we used the Confidence in the Evidence from Reviews of Qualitative Research (CERQual) Tool to assess the evidence for each qualitative finding. Based on the assessment of four components (methodological limitations, relevance, adequacy, and coherence), all qualitative findings were classified into three categories: high confidence, moderate confidence, or low confidence [21].

## Results

**Fig. 1:**
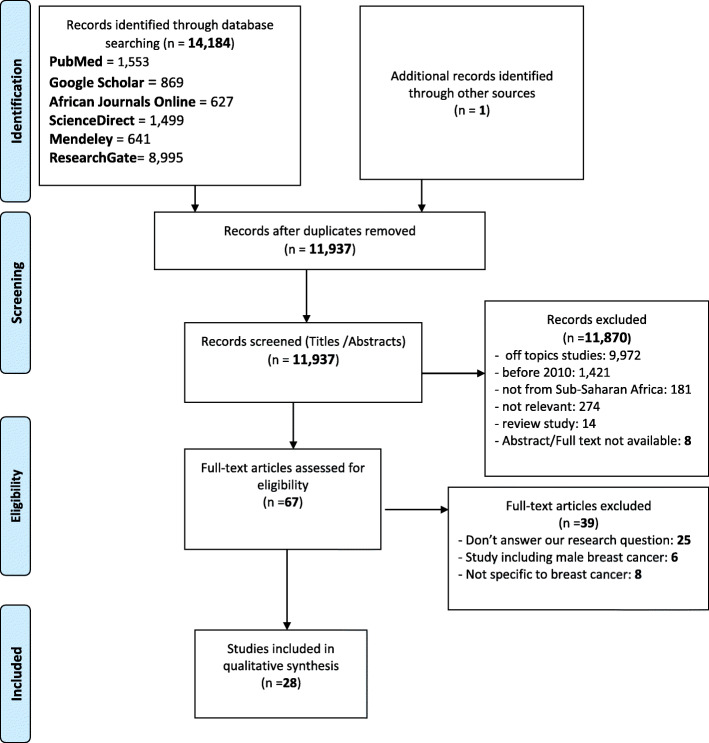
PRISMA diagram flow for studies selection

A total of 14,184 studies were identified by the literature search. After exclusion of duplicate studies and studies outside the scope of our review, only 67 studies were retrieved for full-text review. A total of 28 studies were eligible for inclusion in the review (Fig. 1).

### Study characteristics

The main characteristics of the included studies are summarized in Table 1. Among the 28 studies included in the review, 11 were quantitative, 12 were qualitative, and five were studies with mixed methods (quantitative and qualitative approaches). They were conducted across 13 countries in sub-Saharan Africa. Thirteen (46%) studies were conducted in East Africa, 10 (35%) in West Africa, 3 (10%) were conducted in Southern Africa and 2 (7%) were multi-country studies mixing countries from three parts of sub-Saharan Africa (East, West and Southern Africa). The study publication dates ranged from 2013 to 2020. The sample sizes of the studies ranged from 64 to 1429 for the quantitative and mixed studies. Eleven (69%) quantitative and mixed studies were cross-sectional surveys, while three were cohort studies. The qualitative articles used focus group discussions and in-depth interviews to explore different factors influencing diagnostic and treatment intervals in breast cancer patients. In 21 (75%) studies, their study populations exclusively included women with breast cancer, whether newly diagnosed or not, whereas the seven other studies included physicians, health care workers, family members and women without breast cancer. In the large majority of included studies, women with breast cancer were aged 40 years and over.

**Table 1:** Main characteristics of the included studies

Title	Authors	Year of publication	Country	Study design	Research method	Age group (Years. Median/Mean)	Participants	Sample size	Diagnostic interval	Treatment interval	Diagnostic and treatment intervals	Cited limits	WHO building blocks addressed
(1) Delays in Breast Cancer Presentation and Diagnosis at Two Rural Cancer Referral Centers in Rwanda	Lydia E. Pace et al	2015	Rwanda	Cross-sectional study	Quantitative and qualitative	49	- Women with pathologically confirmed breast cancer	144	5 Months	NA*	NA	- Doubt about the accuracy of the delays noted by patients - Likelihood to not assess all the important contributors to delay - -Included patients might not be representative of all patients with breast cancer in Rwanda - Limited power to detect small differences in patient or system factors that might have been associated with delays	- Service delivery - Health workforce - Information systems
(2) Barriers to early presentation of breast cancer among women in Soweto, South Africa	Maureen Joffe et al	2018	South Africa	Cross-sectional survey	Quantitative and qualitative	> 18	- Female patients who were newly diagnosed with stage 0-IV breast carcinoma	499	NA	NA	NA	- Information about delays in care seeking and numbers and types of healthcare visits is based on patient survey responses. Rather than administrative data. Which may be biased or less accurate - Limited generalizability	- Service delivery - Health workforce
(3) Access to Breast Cancer Treatment Services in Mombasa County. Kenya: A Quality of Care Analysis of Patient and Survivor Experiences	Sultane Sherman and Vincent Okungu	2018	Kenya	Cross-sectional descriptive study	Quantitative and qualitative	36–55	- Breast cancer patients and survivors	72	NA	NA	NA	- The study was conducted in an organized group of cancer survivors and patients and their level of access to services may not entirely reflect the realities of the county where other patients survive without support systems	- Service delivery - Health workforce - Health financing
(4) Factors associated with time to first healthcare visit. Diagnosis and treatment, and their impact on survival among breast cancer patients in Mali	Grosse Frie K et al	2018	Mali	Prospective cohort study	Quantitative and qualitative	45 (Mean)	- Female patients with Breast Cancer	64	6.4 months (mean)	2.5 months (mean)	NA	- Relatively small sample size - This study did not include breast cancer patients who did not go to the hospital for diagnostic services - Women who were lost after the first healthcare visit or never sought healthcare were unknown - These limitations effect the generalizability of the results	- Service delivery - Health workforce
(5) Financial barriers related to breast cancer screening and treatment: A cross-sectional survey of women in Kenya	Subramanian S et al	2019	Kenya	Cross-sectional survey	Quantitative and qualitative	46.1 (Mean)	- 400 Women with breast cancer - 400 Women without a diagnosis of breast cancer	800	NA	NA	NA	- Women were interviewed by research assistants. Which could have also introduced bias - The analysis was based on responses provided by the women themselves. Which may be subject to recall or other biases - All women who participated in this cohort study were identified from the regions within or in close proximity to Nairobi County therefore our findings might not be generalizable - Non-random selection process could have introduced selection bias - Small sample of women with private insurance limited the ability to conduct a stratified analysis to evaluate potential differences by type of insurance coverage	- Health financing - Medication access
(6) Factors linked to late diagnosis in breast cancer in sub-Saharan Africa: Case of Côte d’Ivoire	M. Toure et al	2013	Ivory Coast	Retrospective study	Quantitative	42 (Median)	- Female patients with breast adenocarcinoma	350	< 6–14 Months	NA	NA	NA	- Service delivery - Health workforce - Health Financing
(7) Financial barriers to utilization of screening and treatment services for breast cancer: an equity analysis in Nigeria	Okoronkwo IL et al	2015	Nigeria	Descriptive study	Quantitative	34.69 (Mean)	- women with breast cancer	270	NA	NA	NA	- The respondents were recruited from one hospital only and therefore the study cannot be generalized	- Health Financing
(8) From symptom discovery to treatment - women’s pathways to breast cancer care: a cross-sectional study	Moodley J. et al	2018	South Africa	Cross-sectional study	Quantitative	54	- Women with breast cancer	201	28 days (median)	37 days (median)	NA	- Low recruitment of population targets due to logistic constraints - The timing of interviews could have resulted in difficulty in putting the journey into perspective if women were not emotionally prepared - Interviews conducted in a hospital setting could have resulted in a social desirability bias with under-reporting of time delays - Time intervals reported are unlikely to be representative of intervals seen in public sector settings in SA without a tertiary centre based one-stop breast clinic - Retrospective recall could have affected accurate reporting of symptoms and health seeking behaviours in this study	- Service delivery
(9) Prevalence and Factors Contributing to Late Diagnosis of Breast Cancer among Women Attending Tikur Anbessa Specialized Hospital. Oncology Unit, Addis Ababa, Ethiopia. 2017	Bedada T et al	2018	Ethiopia	Cross-sectional study	Quantitative	41.6	- Newly diagnosed and on-follow-up female breast cancer patient	215	NA	NA	NA	NA	- Service delivery - Health workforce
(10) Educational Opportunities for Down-Staging Breast Cancer in Low-Income Countries: An Example from Tanzania	Yang K et al	2019	Tanzania	Cross- sectional study	Quantitative	51.6	- Female breast cancer patients. 18 years of age and older. Newly referred to Ocean Road Cancer Institute (ORCI) for treatment	196	NA	NA	NA	- Possible recall bias - The medical records did not contain information regarding the previous primary care visits that may have been made	- Service delivery - Health workforce
(11) Time intervals experienced between first symptom recognition and pathologic diagnosis of breast cancer in Addis Ababa. Ethiopia: a cross-sectional study	Gebremariam A. et al	2019	Ethiopia	Cross-sectional study	Quantitative	44.4 (Mean)	- women newly diagnosed with breast cancer	441	2.3 (median)	NA	NA	- The retrospective nature of collecting information about dates of symptom recognition and medical consultations is prone to recall bias - The interviews were conducted in a hospital setting. Which could have resulted in a social desirability bias leading to under-reporting of time interval before medical consultation and over-reporting of desirable behaviours such as self-breast examination	- Service delivery - Health workforce
(12) Impact of Primary Care Delay on Progression of Breast Cancer in a Black African Population: A Multicentered Survey	Agodirin O. et al	2019	Nigeria	Cross-sectional study	Quantitative	48.35 (Mean)	- Breast cancer patients	237	NA	NA	NA	- Although measures were taken to reduce recall bias in the study design. This survey was still limited in that triangulation with the primary care records was impossible because of poor record keeping.	- Service delivery - Health workforce
(13) Inequities in Breast Cancer Treatment in sub-Saharan Africa: Findings from a Prospective Multi-Country Observational Study	Foerster M. et al	2019	-Namibia -Nigeria -Uganda	Prospective Multi-Country Observational Study	Quantitative	50.7 (Mean)	- Women newly diagnosed with breast cancer	1335	NA	NA	NA	- Difficult to disentangle whether untreated proportions were attributable to the country or the specific hospital setting in this specific country - Breast cancer patients who do not reach this level of the health system. and may be more likely to go untreated were not included	- Service delivery - Health financing - Medication access and technologies
(14) Barriers to timely surgery and early surgical outcomes for breast cancer patients in a setting with limited resources	F. Ntirenganya	2019	Rwanda	Cross-sectional study	Quantitative	48 (Median)	Patients who underwent surgery for breast cancer	69	NA	NA	3 months (Mean)	-All patients received standard and adequate therapy according to their diagnosis and staging -The study was conducted in 2 hospitals. With 2 different surgical teams. This can constitute a bias and influence the outcomes	- Service delivery
(15) Presentation Intervals and the Impact of Delay on Breast Cancer Progression in a Black African Population	Agodirin O et al	2020	Nigeria	multicentred survey	Quantitative	50.6 (Mean)	Female breast cancer patients who were newly diagnosed	420	NA	NA	NA	-This Study was limited in that the primary outcome was patient-reported; hence it might be influenced by recall bias	- Service delivery - Health workforce
(16) Dissecting the journey to breast cancer diagnosis in sub-Saharan Africa: Findings from the multicountry ABC-DO cohort study	Foerster M et al	2020	-Namibia -Nigeria -Uganda -Zambia	Prospective multi-country observational study	Quantitative	50.1 (Mean)	women aged ≥18 years with histologically confirmed or suspected breast cancer	1429	NA	NA	NA	-Weaknesses include the fact that participants were recruited in public tertiary referral centres and. Thus. might be unrepresentative as not all breast cancer patients are referred to these hospitals or can reach them -The self-reported length of the diagnostic journey might have been affected by between-woman variation in the ability to recognize symptoms across settings	- Health financing - Health workforce - Service delivery
(17) Social barriers to diagnosis and treatment of breast cancer in patients presenting at a teaching hospital in Ibadan, Nigeria	Pruitt L et al	2014	Nigeria	Qualitative study	Qualitative	51 (Median)	31 women with a diagnosis of breast cancer 5 physicians	36	NA	NA	NA	-This study did not capture the experiences of patients who never made it to tertiary care for breast cancer treatment -The medical setting may also have reduced willingness to speak about complementary and alternative medicine for fear of being judged by their physicians or the belief that such an admission might affect their treatment -Follow-up questions were limited by the use of a translator	- Service delivery - Health workforce - Health financing - Medication access and technologies
(18) Breast Cancer Diagnosis and Factors Influencing Treatment Decisions in Ghana	Aziato. L. and Clegg-Lamptey	2014	Ghana	Qualitative descriptive	Qualitative	31–60 years	Women diagnosed with breast cancer who had undergone a single or bilateral mastectomy	12	NA	NA	NA	NA	- Health workforce - Service delivery
(19) “My experience has been a terrible one. Something I could not run away from”: Zambian women’s experiences of advanced breast cancer	‘Johanna E. Maree And J. Mulonda	2015	Zambia	Qualitative descriptive	Qualitative	48.2 (Mean)	Women living with advanced breast cancer	10	NA	NA	NA	-This was a qualitative study, and no other study reflects on the only true meaning of the narratives, as there could be more than one interpretation -Women who were recruited received treatment at the same hospital	- Health workforce
(20) Understanding pathways to breast cancer diagnosis among women in the Western Cape Province. South Africa: a qualitative study	Jennifer Moodley et al	2016	South Africa	Qualitative	Qualitative	52 (Mean)	Patients with newly diagnosed breast cancer	20	3 Months (Average)	NA	NA	-This study was conducted at one clinic in the Western Cape Province and this limits its generalizability -Women who did not access tertiary healthcare were not included	- Health workforce
(21) A framework for improving early detection of breast cancer in sub-Saharan Africa: A qualitative study of help-seeking behaviors among Malawian women	Kohler Racquel E. et al	2017	Malawi	Qualitative	Qualitative	47 (Median)	Female breast cancer patients	20	NA	NA	NA	-Many Malawian women with breast cancer may never reach a referral hospital (where patients were recruited) -Some women initially experienced symptoms or were diagnosed a few years prior to being interviewed. Therefore, their recollection of events may not be as sharp	- Service delivery - Health workforce - Medication access and technologies
(22) Why Do Women with Breast Cancer Get Diagnosed and Treated Late in Sub-Saharan Africa? Perspectives from Women and Patients in Bamako. Mali	Grosse Frie K et al	2018	Mali	Qualitative study	Qualitative	48 (Mean)	8 women with breast cancer 17 women without breast cancer	25	NA	NA	NA	-Only a small number of women were analysed -There might be further barriers. Particularly for women living outside the capital city. Bamako -Furthermore. experiences and opinions of healthcare personnel and doctors should be researched. as they might balance the views of patients and women	- Service delivery - Health workforce - Medication access
(23) Understanding the causes of breast cancer treatment delays at a teaching hospital in Ghana	Sanuade OA et al	2018	Ghana	Qualitative study	Qualitative	40–49 (modal age range)	Female breast cancer patients	20	NA	NA	< 1–3+ (Min-Max)	- The main limitation of this study was that the number of participants included in the focus groups was very limited	- Service delivery - Health workforce - Health financing
(24) Perceived Barriers to Early Detection of Breast Cancer in Wakiso District. Uganda Using a Socioecological Approach	Ilaboya D et AL	2018	Uganda	Qualitative study	Qualitative	NA	-Woman who have experience in healthcare delivery or health research in relation to cancer care -Community health workers -Key informants	24	NA	NA	NA	-This study involved only one sub-county; therefore, the results may not necessarily be generalizable	- Governance/Leadership - Service delivery - Health workforce
(25) Fear of Mastectomy Associated with Delayed Breast Cancer Presentation Among Ghanaian Women	Martei YM et al	2018	Ghana	Qualitative Study	Qualitative	47.12 (Mean)	Women with a confirmed breast cancer diagnosis	31	NA	NA	NA	- Purposive sampling Only women seen at study site were interviewed - Most of the women seen in the public sector are of lower socioeconomic status, therefore the information may not be generalizable - Financial barriers to presentation and management could be even more significant than those reported in this study	- Health financing - Medication access
(26) Identifying Barriers and Facilitators to Breast Cancer Early Detection and Subsequent Treatment Engagement in Kenya: A Qualitative Approach	Robai Gakunka et al	2019	Kenya	Qualitative	Qualitative	30–60 years	6–11 women with breast cancer 6–11 women without breast cancer	2 Focus groups (6–11 per group)	NA	NA	NA	-Study was carried out in Nairobi and its environs where most of the cancer care services in Kenya are found and, Therefore, it may not be generalizable	- Health financing - Service delivery - Health workforce - Medication access
(27) Perspectives of patients, family members, and health care providers on late diagnosis of breast cancer in Ethiopia: A qualitative study	Gebremariam A et al	2019	Ethiopia	Qualitative study	Qualitative study	< 40 (Modal age range)	13 breast cancer patients 5 family members 5 health workers	23	NA	NA	NA	Recall of past events with the foresight of experience may unconsciously make the stories of these women biased and inaccurate explanations of their experience	- Service delivery - Health workforce - Health financing - Medication access and technologies
(28) Perceived barriers to early diagnosis of breast Cancer in south and southwestern Ethiopia: a qualitative study	Getachaw S et al	2020	Ethiopia	Qualitative study	Qualitative study	26–65 (Modal age range)	12 Breast cancer patients 13 care providers	25	NA	NA	NA	This study used only in-depth interviews for data collection with a limited number of participants	- Governance/Leadership - Service delivery - Health workforce - Health financing - Medication access and technologies

NA* = Not Available

### Factors influencing diagnostic and treatment intervals in breast cancer patients

There was a total of 36 barriers and eight facilitators identified across all studies. Factors identified in each study and classified according to the WHO Health Systems Framework’s six building blocks are summarized in Tables 2 and 3, respectively**.** Among the barriers, misdiagnosis was the most common (16 studies), followed by provider attitude (11 studies) and the high cost of investigation and treatment (11 studies). The appearances of other barriers and facilitators ranged from one to seven times.

**Table 2:** Main factors (barriers and facilitators) identified across studies

Author	Main finding				WHO building blocks addressed
	Quantitative research Barriers Facilitators		Qualitative research Barriers Facilitators		
Lydia E. Pace et al. 2015	- Delayed Referral	NA	- Delayed referral - Delayed Administrative procedures (transfer form) - Provider misinformation	**NA**	- Service delivery - Health workforce - Information systems
Maureen Joffe et al. 2018	- Delayed Referral ( - Delayed appointment or test results - Misdiagnosis	NA	NA		- Service delivery - Health workforce
Sultane Sherman and Vincent Okungu 2018	NA	NA	- Long waiting periods to see an oncologist - Need to travel long distances to get diagnosis and treatment services - Lack of specialist service - Persistent breakdown of radiotherapy machines - High cost of treatment and lack of insurance	**NA**	- Service delivery - Health financing - Technologies - Governance
Grosse Frie K et al. 2018	- Facility and type of medical doctor at the first healthcare facility visited: community care centre or a generalist - No diagnosis or misdiagnosis - Having no health insurance	Being referred by by an oncologist or surgeon	NA	**NA**	- Service delivery - Health workforce - Health financing
Subramanian S et al. 2019	- Hight cost of care and treatment - Lack of insurance - Insurance covered less than expected - Financial impacts due to breast cancer and treatment	NA	- Unavailability of drug - High cost of cancer treatment	NA	- Health financing - Medication access
M. Toure et al. 2013	- Lack of Financial resources - Misdiagnosis - Lack of therapeutic care - Long wait for biopsy results	NA	NA	NA	- Service delivery - Health workforce - Health financing
Okoronkwo IL et al. 2015	- High cost of medical treatment - Lack of health insurance coverage	NA	NA	NA	- Health financing
Moodley J. et al. 2018	- 4 or more healthcare visits between symptom discovery and a breast cancer diagnosis - Long wait for surgery	NA	NA	NA	- Service delivery
Bedada T et al. 2018	- Long waiting time in the reception area - Long waiting time to see a doctor -Unavailability of an appropriate doctor --Inappropriate diagnosis - No imaging investigations available - Professional’s lack of appropriate attention - Professional’s inability to examine the patient appropriately (patient’s perception)	NA	NA	NA	- Service delivery - Health workforce
Yang K et al. 2019	- Hospital’s failure to inform patient of biopsy requirements - Delayed Referral - Difficulty with navigating the referral system - Lack of knowledge by provider - Healthcare professional’s misinterpretation of biopsy results - Inappropriate treatment - No referral for further care upon initial presentation - Misdiagnosis - Biopsy results delayed	NA	NA	NA	- Service delivery - Health workforce
Gebremariam A. et al. 2019	- Misdiagnosis - False-negative laboratory results - Lack of empathy at first medical consultation - Visited ≥4 different healthcare facilities before diagnostic confirmation	- Visited a public hospital at the first consultation	NA	NA	- Service delivery - Health workforce
Agodirin O. et al. 2019	- Delayed Referral (long primary care interval for 69.3% patients) - Long distance to the specialist clinic - Visiting more than one provider before diagnosis confirmation - Misinformation (incorrect advice or directive from first healthcare provider) - Misdiagnosis and mistreatment (first healthcare provider error) - Awaiting results - Conflicting results - Difficult navigation - Strike	NA	NA	NA	- Service delivery - Health workforce - Governance
Foerster M. et al. 2019	- Expensive healthcare - Cost of surgery - Healthcare expenses paid out-of-pocket by the patient - Equipment (radiotherapy) not available	- Have a healthcare coverage - availability of free health care -Availability of equipment	NA	NA	- Health financing - Medication access and technologies
F. Ntirenganya 2019	-Long waiting for transfer to health facility offering breast cancer surgery - Long waiting for consultation by a surgeon - Long waiting for biopsy results - Long waiting for imaging/staging investigations	NA	NA	NA	- Service delivery - Health workforce
Agodirin O et al. 2020	- Misdiagnosis by first healthcare provider - Delayed Referral and long primary care interval - Inappropriate reassurance by first healthcare provider - Strike - Mistrust in conventional medicine	NA	NA	NA	- Service delivery - Health workforce
Foerster M et al. 2020	- Misdiagnosis - Inappropriate reassurance - Visits to 1 to 4 healthcare providers before diagnostic hospital - High Treatment costs	NA	NA	NA	- Health financing - Health workforce
Pruitt L et al. 2014	NA	NA	- Inappropriate medical care (non-physician community healthcare provider) - Long waiting for test results - Strikes by hospital staff - Long waiting for surgery scheduling - High costs of treatment - Default histologies and communication	NA	- Service delivery - Health workforce - Health financing - Medication access and technologies
Aziato. L. and Clegg-Lamptey 2014	NA	NA	- Misdiagnosis - Long waiting for biopsy results	NA	- Health workforce - Service delivery
Johanna E. Maree And J. Mulonda 2015	NA	NA	- Misdiagnosis - Mismanagement	NA	- Health workforce
Jennifer Moodley et al. 2016	NA	NA	- Misdiagnosis	NA	- Health workforce
Kohler Racquel E. et al. 2017	NA	NA	- Poor provider knowledge and misdiagnosis - Poor delivery processes - Medical equipment failure - Poor access to providers and service - Long waiting for biopsy results - Delayed Referral - Unavailability of medication and provider channels - Lack of provider communication	NA	- Service delivery - Health workforce - Medication access
Grosse Frie K et al. 2018	NA	NA	- Misdiagnosis - Wrong medication prescription - Mistrust in healthcare workers - Unavailability of doctors or drugs	NA	- Service delivery - Health workforce - Medication access
Sanuade OA et al. 2018	NA	NA	- High cost of chemotherapy pharmaceutical drugs and other associated costs of breast cancer treatment - Healthcare workers’ attitude corruption - Wrong/harmful advice to patients by encouraging them to seek alternative treatment - Long queues during treatment - Unavailability of doctors - Breakdown of hospital machines - Shortage of medication access - Workload of the doctors - Shortage of healthcare workers - Slow moving queues at the drug dispensary - Delayed biopsy results from the pathology department - Long distance between departments involved in breast cancer treatment within the hospital premises	NA	- Service delivery - Health workforce - Health financing
Ilaboya D et al. 2018	NA	NA	- Lack of training and lack of breast cancer knowledge among community health workers - Low prioritization of NCDs - Lack of cancer policy - Lack of cancer services at the primary healthcare level - Geographical inaccessibility of health facilities	NA	- Governance/Leadership - Service delivery - Health workforce
Martei YM et al. 2018	NA	NA	- Lack of financial resources - High cost of chemotherapy drugs - Limited insurance coverage for chemotherapy and radiation treatment	NA	- Health financing - Medication access
Robai Gakunka et al. 2019	NA	NA	- Inadequate insurance coverage - Expensive private insurance - Discrimination by private insurers - Misdiagnosis - Poor communication by caregivers about diagnosis and financial implications causing mistrust between patients and caregivers - High cost of care	- Short waiting period - Drug availability - Good communication by healthcare givers	- Health financing - Service delivery - Health workforce - Medication access
Gebremariam A et al. 2019	NA	NA	- Physicians misunderstanding of the first symptom - Inappropriate reassurance that the lump is benign without biopsy - Long waiting times to receive diagnostic confirmation - Few diagnostic centres - Poor provider-patient communication and counselling - High costs of investigation and treatment - Delayed referral - Long waiting period for consultation	NA	- Service delivery - Health workforce - Health financing - Medication access and technologies
Getachaw S et al. 2020	NA	NA	- High treatment costs - delayed care transitions - Poor provider knowledge - Misdiagnosis - Inappropriate treatment - Delayed Referral - Long distance to referral facilities - Lack of clinical breast examination practice by provider - Delayed Appointment - Poor attention by provider - Inadequate examinations - Poor communication between healthcare providers and patients - Several visits to health facilities to get their diagnosis - High cost of diagnostic services - Long waiting time for diagnostic tests - Lack of screening and diagnostic tests in local facilities - Lack of health education programmes and skilled professionals	NA	- Governance/Leadership - Service delivery - Health workforce - Health financing - Medication access and technologies

**Table 3:** Factors (barriers and facilitators) classified according to the WHO building blocks

WHO building blocks	Factors identified	
	**Barriers**	**Facilitators**
**Service delivery**	- Delayed test results - Delayed appointment - Delayed referral and long primary care interval - Decreased access to providers and services - Poor delivery process - Long wait for surgery/treatment - One to ≥4 or more healthcare visits between symptom discovery and a breast cancer diagnosis - Long waiting time in hospital reception - Difficulty navigating referral system - Long waiting for imaging/staging investigation - Long waiting for transfer to health facility offering breast cancer surgery - Long queue during treatment and drug dispensation - Lack of cancer service in primary care - Geographical inaccessibility/long travel distance - Few diagnostic centres - Long waiting times for diagnostic confirmation	- Be reffered by an oncologist or surgeon - Visited a public hospital at the first consultation Short waiting period
**Health workforce**	- Misdiagnosis - Mismanagement - Provider misinformation - Provider’s poor attitude - Lack of knowledge among providers - Lack of providers training - No appropriate physician/unavailability of doctors - Strike	- Good communication by healthcare providers
**Information system**	- Delayed administrative procedures	NA
**Health financing**	- High cost of treatment/investigations - Lack of insurance - Limited insurance coverage - Expensive private insurance - Discrimination by private insurance - Financial impact of breast cancer treatment	- Have a healthcare coverage - availability of free health care
**Medication access and technologies**	- Persistent breakdown of hospital machines/medical Equipment failure - Shortage of medicine/unavailability of drug - Lack of screening and diagnostic equipment in local facilities	- Availability of equipment - Drug availability
**Governance/Leadership**	- Lack of cancer policy - Low prioritization of NCDs	NA

#### Health service delivery

Health service delivery was addressed by 23 of the studies included in the review, and the factors identified can be grouped into two major themes: logistics and infrastructure.

Logistical difficulties included different waiting times for an appointment (medical or specialist consultation), investigations (imaging, biopsy), test results, referral or treatment (surgery, radiotherapy) [22–30]. A long waiting time for test results, more specifically for the biopsy results, was a factor influencing diagnostic and treatment intervals in 11 included studies [23, 25–29, 31–35]. In seven studies included in this review, visiting other health facilities (1 to 4 times or more) before attending the breast cancer diagnostic center was identified as one of the reasons for longer diagnostic intervals [22, 23, 29, 35–38]. Studies have also shown that the type of healthcare facility and the type of health worker visited at the first consultation had an impact on diagnostic and treatment intervals [38, 39], and women who first visited a community health centre or general practitioner experienced longer time intervals than those who first saw a specialist (surgeon or oncologist) [38, 39]. Being referred by an oncologist or surgeon or having received the first consult evaluation in a public hospital have been identified as factors facilitating diagnosis and access to treatment [38, 39]. Being referred directly to tertiary care hospital with specialized services after the first visit to the primary care clinic or general practitioner while bypassing a secondary care hospital (without specialized services) was identified as a factor facilitating women’s access to diagnosis and treatment. Indeed, women who went through the secondary care hospital or other health facilities were more likely to experience long diagnostic intervals [23, 40]. Logistic problems also included poor organization and unavailability or shortage of breast cancer services [29, 30, 41].

The geographical inaccessibility of healthcare facilities and the insufficiency of diagnostic centres are part of the infrastructure problems [27–30, 41]. In Uganda, for instance, one patient interviewed said, *‘The health centre nearby the community does not offer screening services, and someone may find it hard to leave this place [Ssisa sub-county] to go to Kampala; but if they bring the services closer to the community, some will find it easier to visit them.’* [Semi-structured #2] [41].

#### Health workforce

##### Misdiagnosis, misinterpretation and mismanagement

Among health workforce factors, misdiagnosis was the most common and appeared in 16 studies [23, 24, 28, 29, 31, 33–35, 37–39, 42–46]. Patients reported being inappropriately reassured by health workers that their breast lump was benign without a biopsy or with an incorrect biopsy interpretation.

“*… I noticed something, a small lump on my breast … I woke up in the morning and went to my doctor. He told me it could be a tumour. I asked him if it could be a cancer because I heard about it on TV. He told me it is not a cancer.” (P07)* [28].

Symptom misinterpretation and misdiagnosis were the most frequent reasons for prolongation of the primary care interval. In six studies, caregivers mismanaged breast cancer patients by giving incorrect medical prescriptions or incorrect advice [26, 27, 29, 35, 43, 44]. For instance, Pruitt et al. reported that the majority of women described receiving oral medication or injections, usually antibiotics, sometimes for months or years before being referred or making an independent decision to seek care elsewhere [26].

*‘I went to the Referral Hospital in XXX. They gave me drugs which I took for 3 months. During that period, I did not see any amelioration … 3 months later I went back to my doctor and he gave me other prescriptions for blood analyses … ‘(Patient 7*) [44].

Olayide Agodirin et al. found that the rate of long primary care intervals was higher among patients who received incorrect advice (81%, 44 of 54) than among those who received correct advice (67%, 100 of 148) (OR 2.1, 95% CI 1.0–4.6) [35].

##### Poor knowledge and skills

Misdiagnosis is often attributed to poor knowledge and lack of health workers training about breast cancer; most health workers provided incorrect information to patients [24, 29, 31, 34, 41]. For instance, in one study, women reported that some of the health workers were unable to examine them appropriately [24]. As an illustration, in one focus group, a health worker said: *‘For me, I have never gotten training on breast cancer detection but I just hear that breast cancer is very dangerous and it is good for someone to go for check-ups but I have never received training on breast cancer examination.’ [FGD CHWs #7]* [41]*.*

##### Attitude of health workers

The attitude of providers was also an important factor influencing women’s access to treatment. Some studies reported that poor attitudes and corruption among health workers were factors that accounted for delays in the start of definitive treatment [27]. Sanuade et al. reported that women in Ghana experienced longer treatment intervals due to corrupt practices of health workers favoring patients they knew personally. Other women said they used bribery to alleviate delays in accessing treatment [27]. Patients also said some health workers disrespect them, refused to answer their questions or did not treat them well, which forced them to delay the start of treatment [27], while others had good communication with health care providers, which made it easier for them to manage their breast cancer [46].

##### Strikes and shortage of caregivers

The shortage of health workers and strikes were also identified as factors delaying women’s access to breast cancer treatment [24, 26, 27, 31, 35, 42, 44]. Studies have reported that various hospital departments turned women away because a doctor was not available [31]. Women faced a long time intervals in receiving their test results and had difficulty accessing care due to strikes by various members of the healthcare team, including consultants and residents [26].

#### Health financing

Six key elements were identified among financing-related factors: high costs of treatment and investigations, lack of insurance or limited insurance coverage, expensive private insurance and discrimination by private insurance. For instance, some of the breast cancer patients were often not eligible for private health insurance [47]. The high cost of treatment was reported as an important factor influencing women’s access to breast cancer treatment in 11 studies [26–30, 37, 46–50]. One woman in the study reported by Sanuade et al. said, *“The chemo is expensive. The trauma and money you spend is a problem too. If you do not have at least 200 Ghana cedis, you cannot buy the drugs. When someone hears all this, the individual would opt for herbal medicine or prayer. So, as for me, I think that lack of money is a factor. I paid 1000 Ghana cedis to use the chemo machine. If you do not have money, you would go home. So, money is a serious factor.” (FGD 4-R1)* [27]*.*

Lack of or limited insurance coverage was also mentioned as a barrier to care. In the study conducted by Subramanian et al. in Kenya, 78% out of 400 women with breast cancer reported borrowing money from family or friends to cover out-of-pocket medical and related expenses [47]. In another study conducted in the Ivory Coast, 36% out of 126 patients declared having had a delayed diagnosis due to lack of financial resources [33]. Many women reported having no insurance coverage [47–49]. For instance, Okoronkwo et al. found that 71.8% out of 194 patients studied in Nigeria did not have health insurance coverage [48].

#### Medication access and technologies

Unavailability of drugs and equipment failure were the most common factors among those related to medication access and technologies [27, 29, 31, 44, 46, 47]. Diagnostic assessments were not available in small health centres, and multiple visits were required for X-rays or blood tests or to obtain a biopsy sample, according to interviewees. Various hospital departments turned women away because the computer was broken or the X-ray machine was not working [27, 29, 31]. Study results also identified drug shortages as an important factor in lengthening the treatment time interval [31, 44, 46].

For instance, key informants in one study conducted in Uganda reported that the existing health system is not equipped to manage breast cancer [41].

#### Leadership and governance

Lack of cancer policies and low prioritization of non-communicable diseases, were the common factors identified in the included studies [41]**.** Key informants in one study conducted in Uganda highlighted the lack of cancer policy-providing guidelines for cancer management across each spectrum of the cancer care continuum [41].

*‘There is no such policy on cancer screening or cancer prevention; there’s nothing like that.’* [*key Informant #6*] [41].

#### Information system

The information system was addressed by only one study among those included in the review [36]. The key factor identified was the delay in administrative procedures. In this study, conducted in Rwanda, 27% out of 113 women interviewed said that, to receive public insurance coverage for care provided at a district hospital, a referral form needs to be signed by the referring health center. Many patients described the need for a transfer form as a reason for the delay.

### Quality appraisal of the included studies

The majority of the quantitative studies were rated as good quality based on the NIH study quality assessment tools for Observational Cohort and Cross-Sectional Studies **(**Supplementary data, Table S1). Most qualitative studies were of high quality based on the CASP checklists **(**Supplementary data, Table S2**)**. There was no low quality among studies according to the individual assessment of studies based on the NIH Study Quality Assessment Tools and CASP Checklists.

Based on the four components (methodological limitations, relevance, adequacy, and coherence) of the CERQual approach, the confidence in the majority of the qualitative evidence summated (16 of 21) was rated as low. (Supplementary data, Table S3).

## Discussion

This review aimed to identify the health system factors that influence diagnostic and treatment intervals in women with breast cancer in sub-Saharan Africa. The qualitative synthesis of studies identified 44 factors, including 36 barriers and 8 facilitators. These factors are mainly related to health service delivery, health workforce and financing, followed by factors related to medication access and technologies, governance and leadership, and the information system.

Poor organization of health service delivery was responsible for increasing the waiting times for different investigations (often the biopsy, consultation or surgery appointment, and referrals). This was mainly attributed to misdiagnosis, mismanagement or misinterpretation and a long primary care interval. The geographical inaccessibility of different services was also found to be an important barrier to care. These results may be a reasonable representation of the phenomenon of interest according to the level of confidence in the qualitative evidence, as assessed by the CERQual tool. However, they are supported by quantitative and mixed studies that were mostly assessed as good quality studies. These results are consistent with those reported in a recently published systematic review by Nathan R. Brand et al. in LMICs. In their review, 92 studies looked at breast cancer, 10 identified reduced access to primary care, 6 identified limited access to diagnostic services, and 14 identified geographic inaccessibility as factors related to health system [51]. Similar results were reported in studies conducted in Morocco [52–54], Palestine [55] and Brazil [56]. These findings suggest the need for efforts to be deployed by decision-makers to ensure the availability and quality of screening services and specialized and comprehensive care for breast cancer in sub-Saharan Africa.

The factors related to the health workforce have been identified as a major handicap to women’s access to treatment in our review. These are mainly diagnostic errors and inadequate care, especially at first contact with the health system, followed by the attitude of providers and the lack of human resources. According to the CERQual assessment tool, these data provide a moderate level of confidence in the synthesis of the qualitative findings, and they are also found in quantitative and mixed studies of good methodological quality according to the NIH Assessment Tool. In addition, it is important to note the variations in some attitudes of health professionals that were identified by these studies, particularly in terms of communication, trust, patient information and corruption. These variations would be related to the differences in contexts and experiences lived and reported by women. However, inappropriate diagnosis remains the most common factor in our studies and is also found in other settings. In their critical review, Unger-Saldaña K. identified medical errors in initial diagnosis, screening interpretation and pathology review as factors related to access or quality of care deficiencies that have been associated with diagnostic and treatment intervals in different countries, such as the United States, England Thailand, Scotland, Netherlands, Canada and Mexico [9]. Our results are also in line with those of the review conducted in Africa by Espina et al. [11] These very alarming findings should arouse enormous interest among professionals in health care and health policies to give importance to initial and continuing medical training in breast cancer.

As a factor related to financing, this review identified the probable influence of treatment costs. Lack of health insurance or limited health insurance coverage forces women to pay for services out of pocket, which is not always easy for the most vulnerable individuals. These results are consistent with those of a review conducted in the Middle East and North Africa (MENA) region [57] and with a similar study conducted in India [58]. The financing-related factors identified in our review have been reported by mostly high-quality qualitative studies according to the CASP Assessment Tool and good-quality quantitative studies according to the NIH Assessment Tool. It is therefore necessary to accelerate the race for medical coverage to ensure adequate and timely care for patients, as stipulated in the sustainable development goals (SDGs) adopted by the United Nations General Assembly [59].

The remaining three health system building blocks (medication access and technologies, governance and leadership, and information systems) were weakly represented. This is likely the influence of the lack of health policies and the possible influence of other factors, such as the lack or shortage of drugs, care equipment failure and the low prioritization of non-communicable diseases. The only factor related to the information system is the difficulty of administrative procedures. This factor was reported in one mixed study of good methodological quality. The relevance of factors related to medication access and technologies, governance and the information system remains limited. Therefore, more primary studies on these factors and, above all, adapting health policies to local specificities should be recommended.

The few facilitators identified in this review reflect a variation and inequity in women’s access to care between some health structures but also between countries in sub-Saharan Africa. These facilitators were related to free health care, sufficient health coverage and the availability of resources. In a review conducted in the MENA region, having a health insurance facilitated access to mammography [57].

Studies that reported these same factors in high-income countries are mostly more than 10 years old [60, 61]. This would indicate two major conclusions: first, our phenomenon of interest is no longer a real problem for them, and second, there has been a clear advancement in the management of women with breast cancer in high-income countries compared to low- and middle-income countries. However, recent studies conducted in these countries have shown inequalities in access to breast cancer diagnosis and care for vulnerable populations such as immigrant women, residents of rural areas and black individuals [62–64].

### Strengths and limitations of this review

To our knowledge, this is the first systematic review focused on health system factors that influence diagnostic and treatment intervals in women with breast cancer in sub-Saharan Africa. The majority of the included studies were of good methodological quality. Our review also has several limitations. First, the non-inclusion of grey literature does not exclude the risk of publication bias. Second, the review included studies from only 13 of the 48 sub-Saharan African countries, and the phenomenon studied could be worse or better for countries in which there are no data available. This reveals the paucity of published data on this topic, limits the relevance of our results and suggests the need for more primary research on the topic in the region. Third, most included studies explored more than 5000 women with breast cancer at the hospital level, which could constitute a selection bias. In fact, only women who succeeded in accessing healthcare facilities and obtaining a diagnosis were enrolled in the included studies, further limiting the representativeness or generalizability of the data. This limitation has been underlined by several of the included studies. The heterogeneity noted in the definition and quantification of the different time intervals did not affect the importance of the factors identified or hinder our initial objective, which was to identify any factor that could influence women’s access to diagnosis and treatment.

### Implications for health policies and health system research

Despite the methodological limitations identified in some studies, our review findings suggest the need for developing appropriate breast cancer policies that take into account the reduction of financial and geographical accessibility barriers, the strengthening of the management of health service delivery to ensure the availability, the quality of timely screening services, specialized and comprehensive care for breast cancer in sub-Saharan Africa. Attention should also be paid to the continuing education, formative supervision for frontline health workers.

Evidence from LMICs suggests that there is no single best way to improve timely access for women to breast cancer diagnosis and treatment. In line with Haford et al. and Horton et al. [65–67], we suggest that effective reforms need to be comprehensive and context specific. These reforms need to include the development of National health insurance schemes, National breast cancer control plans (Zambia, Ghana), integration of breast cancer programs within existing health system platforms (Zambia and Tanzania) to benefit from pooled resources.

We also stress the importance of continuing education, formative supervision for frontline and community health workers in line with Pace et al. [68], community participation and the development of patient navigation programs coupled with public advocacy to improve early detection of breast cancer [65, 67].

Our review has also indicated some research gaps such as the importance of exploring the role of medication access and technologies, governance and leadership, and information systems facilitating women’s access to appropriate and timely breast cancer treatment in sub-Saharan Africa.

## Conclusion

Our review indicates that diagnostic and treatment intervals among women with breast cancer in sub-Saharan Africa are influenced by many health system related factors. When women manage to overcome their fear, lack of knowledge, socioeconomic and cultural conditions, they also end up being challenged by overwhelming health system factors that they cannot cope with. Our review sheds light on the underlying factors that explain the longer time intervals and health system challenges women face in terms of financial and geographical access to care, diagnostic errors, inappropriate management and lack of an adequate cancer health policies.

## Supplementary Information

**Additional file 1: Table S1.** NIH Quality Assessment Tool for observational cohort and cross-sectional studies.** Table S2.** Critical Appraisal Skills Program (CASP) Quality-Assessment Tool for qualitative studies. **Table S3.** CERQual assessment of confidence of qualitative findings. **Table S4.** Searches strategies.** Table S5.** List of full-text articles excluded and reasons for exclusion.

## Data Availability

All data generated or analysed during this review are included in this document and its supplementary data.

## Abbreviations

PRISMA: Preferred Reporting Items for Systematic Reviews and Meta-Analyses
PROSPERO: International prospective register of systematic reviews
CASP: Critical Appraisal Skills Program
NIH: National Institute of Health
CERQual: Confidence in the Evidence from Reviews of Qualitative Research
LMICs: Low-and middle-income countries
MENA: Middle East and North Africa
FGD: Focus group discussion
CHWs: Community health workers
SDGs: Sustainable development goals
WHO: World Health Organization
