# Supplementary material for: Health system factors that influence diagnostic and treatment intervals in women with breast cancer in sub-Saharan Africa: a systematic review
Source: BMC Public Health. 2021 Jul 6;21:1325. doi: 10.1186/s12889-021-11296-5 (PMC8259007; doi:10.1186/s12889-021-11296-5)
Supplement: Supplementary file 1 — Additional file 1: Table S1. NIH Quality Assessment Tool for observational cohort and cross-sectional studies. Table S2. Critical Appraisal Skills Program (CASP) Quality-Assessment Tool for qualitative studies. Table S3. CERQual assessment of confidence of qualitative findings. Table S4. Searches strategies. Table S5. List of full-text articles excluded and reasons for exclusion. [file 12889_2021_11296_MOESM1_ESM.docx]

| **Table S1: NIH Quality Assessment Tool for Observational Cohort and Cross-Sectional Studies** | | | | | | | | | | | | | | | |
| --- | --- | --- | --- | --- | --- | --- | --- | --- | --- | --- | --- | --- | --- | --- | --- |
| **Author/Reference** | **Was the research question or objective in this paper clearly stated?** | **Was the study population clearly specified and defined?** | **Was the participation rate of eligible persons at least 50%?** | **Were all the subjects selected or recruited from the same or similar populations (including the same time period)? Were inclusion and exclusion criteria for being in the study prespecified and applied uniformly to all participants?** | **Was a sample size justification, power description, or variance and effect estimates provided?** | **For the analyses in this paper, were the exposure(s) of interest measured prior to the outcome(s) being measured?** | **Was the timeframe sufficient so that one could reasonably expect to see an association between exposure and outcome if it existed?** | **For exposures that can vary in amount or level, did the study examine different levels of the exposure as related to the outcome (e.g., categories of exposure, or exposure measured as continuous variable)?** | **Were the exposure measures (independent variables) clearly defined, valid, reliable, and implemented consistently across all study participants?** | **Was the exposure(s) assessed more than once over time?** | **Were the outcome measures (dependent variables) clearly defined, valid, reliable, and implemented consistently across all study participants?** | **Were the outcome assessors blinded to the exposure status of participants?** | **Was loss to follow-up after baseline 20% or less?** | **Were key potential confounding variables measured and adjusted statistically for their impact on the relationship between exposure(s) and outcome(s)?** | **Overall** |
| Lydia E. Pace et al,  2015 | Yes | Yes | Yes | Yes | Yes | No | NA | NA* | NA | NA | Yes | NA | NA | Yes | Good |
| Maureen Joffe et al,  2018 | Yes | Yes | Yes | Yes | Yes | No | NA | NA | NA | NA | Yes | NA | NA | Yes | Good |
| Sultane Sherman and Vincent Okungu,  2018 | Yes | Yes | Yes | Yes | Yes | No | NA | NA | NA | NA | No | NA | NA | NR | Fair |
| Grosse Frie K et al,  2018 | Yes | Yes | Yes | Yes | Yes | No | Yes | NA | NA | NA | Yes | NA | Yes | Yes | Good |
| Subramanian S et al,  2019 | Yes | Yes | Yes | Yes | Yes | No | NA | NA | NA | NA | NR* | NA | NA | No | Fair |
| M. Toure et al,  2013 | Yes | Yes | Yes | Yes | Yes | No | NA | NA | NA | NA | Yes | NA | NA | Yes | Good |
| Okoronkwo IL et al,  2015 | Yes | Yes | Yes | Yes | Yes | No | NA | NA | NA | NA | Yes | NA | NA | No | Fair |
| Moodley J. et al,  2018 | Yes | Yes | Yes | Yes | Yes | No | NA | NA | NA | NA | Yes | NA | NA | Yes | Good |
| Bedada T et al,  2018 | Yes | Yes | Yes | Yes | Yes | No | NA | NA | NA | NA | Yes | NA | NA | Yes | Good |
| Yang K et al,  2019 | Yes | Yes | Yes | Yes | Yes | No | NA | NA | NA | NA | Yes | NA | NA | Yes | Good |
| Gebremariam A. et al,  2019 | Yes | Yes | Yes | Yes | Yes | No | NA | NA | NA | NA | Yes | NA | NA | Yes | Good |
| Agodirin O. et al,  2019 | Yes | Yes | Yes | Yes | Yes | No | NA | NA | NA | NA | Yes | NA | NA | No | Fair |
| Foerster M. et al,  2019 | Yes | Yes | Yes | Yes | Yes | No | Yes | NA | NA | NA | Yes | NA | NR | Yes | Good |
| F. Ntirenganya,  2019 | Yes | Yes | Yes | Yes | Yes | No | NA | NA | NA | NA | Yes | NA | NA | No | Fair |
| Agodirin O et al,  2020 | Yes | Yes | Yes | Yes | Yes | No | NA | NA | NA | NA | Yes | NA | NA | Yes | Good |
| Foerster M et al,  2020 | Yes | Yes | Yes | Yes | Yes | No | Yes | NA | NA | NA | Yes | NA | NR | Yes | Good |

**NA*: Not applicable; NR*: Not reported**

**Table S2: Critical Appraisal Skills Program (CASP) quality-assessment tool for qualitative Studies**

| Author/ Reference | Was there a clear  statement of the aims of  the research? | Is a qualitative  methodology  appropriate? | Was the research  design appropriate to  address the aims of the  research? | Was the recruitment  strategy appropriate to  the aims of the  research? | Was the data collected in  a way that addressed the  research issue? | Has the relationship  between researcher and  participants been  adequately considered? | Have ethical issues been  taken into consideration? | Was the data analysis  sufficiently rigorous? | Is there a clear statement  of findings? | How valuable is the research? | Overall |
| --- | --- | --- | --- | --- | --- | --- | --- | --- | --- | --- | --- |
| Pruitt L et al  2014 | ⬤ | ⬤ | ⬤ | • | • | ⚫ | • | ⬤ | ⬤ | • | Medium |
| Aziato. L. And Clegg-Lamptey  2014 | ⬤ | ⬤ | ⬤ | ⚫ | ⬤ | ⬤ | ⬤ | • | ⬤ | ⚫ | High |
| Johanna E. Maree  And J. Mulonda  2015 | ⬤ | ⬤ | ⬤ | ⚫ | ⬤ | ⬤ | ⬤ | ⚫ | ⬤ | ⚫ | High |
| Jennifer Moodley et al  2016 | ⬤ | ⬤ | ⬤ | ⚫ | ⚫ | ⚫ | ⬤ | ⬤ | ⬤ | ⬤ | High |
| Kohler Racquel E. et al  2017 | ⬤ | ⬤ | ⬤ | ⚫ | ⚫ | ⚫ | ⬤ | ⚫ | ⚫ | ⬤ | Medium |
| Grosse Frie K et al  2018 | ⬤ | ⬤ | ⬤ | • | ⬤ | ⚫ | ⚫ | ⬤ | ⬤ | ⚫ | High |
| Sanuade OA et al  2018 | ⬤ | ⬤ | ⬤ | • | ⬤ | ⚫ | • | ⬤ | ⬤ | • | High |
| Ilaboya D et aL  2018 | ⬤ | ⬤ | ⬤ | ⚫ | ⬤ | • | ⚫ | ⬤ | ⬤ | ⬤ | High |
| Martei YM et al  2018 | ⬤ | ⬤ | ⬤ | ⚫ | ⬤ | • | • | ⬤ | ⬤ | ⬤ | High |
| Robai Gakunka et al  2019 | ⬤ | ⬤ | ⬤ | ⚫ | ⚫ | ⬤ | ⚫ | ⚫ | ⚫ | ⬤ | Medium |
| Gebremariam A et al  2019 | ⬤ | ⬤ | ⬤ | • | ⬤ | ⚫ | • | ⬤ | ⬤ | ⚫ | High |
| Getachaw S et al  2020 | ⬤ | ⬤ | ⬤ | ⚫ | ⬤ | • | ⚫ | ⬤ | ⬤ | • | High |

⬤ High ⚫ Medium • Low

| **Table S3: CerQual Assessment of confidence of qualitative findings** | | | | | | | |
| --- | --- | --- | --- | --- | --- | --- | --- |
| **Review Finding** | **Studies Contributing to the Review Finding** | **Assessment of Methodological Limitations** | **Assessment of Relevance** | **Assessment of Coherence** | **Assessment of Adequacy of Data** | **Overall CERQual Assessment of Confidence** | **Explanation of Judgement** |
| **Service delivery** |  |  |  |  |  |  |  |
| Waiting test results | 17, 18,21, 23,27,28 | **Moderate methodological limitations** (4 studies with minor concerns, 2 studies with moderate concerns) | **High concerns about relevance** (partial relevance, studies from 4 countries: Nigeria, Ghana, Malawi, Ethiopia) | **Minor concerns regarding coherence** | **High concerns about adequacy** **of data** (limited, thin data from 6 studies) | **Low** | This finding was graded as Low confidence because of Moderate concerns regarding methodological limitations and high concerns about relevance and adequacy of data |
| Waiting treatment | 17 | **Moderate methodological limitations** (1 study with moderate concerns) | **High concerns about relevance** (partial relevance, study from 1 country: Nigeria) | **Extent of coherence unclear due to limited data** | **High concerns about adequacy of data** (limited, thin data from 1 study) | **Low** | This finding was graded as Low confidence because of Moderate concerns regarding methodological limitations and high concerns about relevance and adequacy of data |
| Poor delivery process | 21 | **Moderate methodological limitations (**1 study with moderate concerns) | **High concerns about relevance** (partial relevance, study from 1 country: Malawi) | **Extent of coherence unclear due to limited data** | **High concerns about adequacy of data** (limited, thin data from 1 study) | **Low** | This finding was graded as Low confidence because of Moderate concerns regarding methodological limitations and high concerns about relevance and adequacy of data |
| Access to providers and services | 21,23, 24, 26, 27,28 | **Moderate methodological limitations** (4 studies with minor concerns, 2 studies with moderate concerns) | **High concerns about relevance** (partial relevance, studies from 5 countries: Malawi, Ghana, Uganda, Kenya, Ethiopia) | **Minor concerns regarding coherence** | **High concerns about adequacy** of data (limited, thin data from 6 studies) | **Low** | This finding was graded as Low confidence because of Moderate concerns regarding methodological limitations and high concerns about relevance and adequacy of data |
| Delayed Referral | 21,27,28 | **Moderate methodological limitations** (2 studies with minor concerns, 1 study with moderate concerns) | **High concerns about relevance** (partial relevance, studies from 2 countries: Malawi, Ethiopia) | **Extent of coherence unclear due to limited data** | **High concerns about adequacy** **of data** (limited, thin data from 3 studies) | **Low** | This finding was graded as Low confidence because of Moderate concerns regarding methodological limitations and high concerns about relevance and adequacy of data |
| **Health Workforce** |  |  |  |  |  |  |  |
| Misdiagnosis | 18, 19, 20, 21, 22, 26, 27, 28 | **Minor methodological limitations** (6 studies with minor concerns, 2 studies with moderate concerns) | **High concerns about relevance** (partial relevance, studies from 7 countries: Ghana, Zambia, South Africa, Malawi, Mali, Kenya, Ethiopia) | **Minor concerns regarding coherence** | **Moderate concerns about adequacy of data** (limited, slightly thick data from 8 studies) | **Moderate** | This finding was graded as Moderate confidence because of Minor concerns regarding methodological limitations and coherence, in addition to high concerns about relevance and moderate concerns regarding adequacy of data |
| Mismanagement | 17,19,22,27,28 | **Minor methodological limitations** (4 studies with minor concerns, 1 study with moderate concerns) | **High concerns about relevance** (partial relevance, studies from 3 countries: Nigeria, Zambia, Mali, Ethiopia) | **Minor concerns regarding coherence** | **Moderate concerns about adequacy of data** (limited, slightly thick data from 5 studies) | **Moderate** | This finding was graded as Moderate confidence because of Minor concerns regarding methodological limitations and coherence, in addition to high concerns about relevance and moderate concerns regarding adequacy of data |
| Provider attitude | 17,21,22,23, 26,27,28 | **Moderate methodological limitations** (4 studies with minor concerns, 3 studies with moderate concerns) | **High concerns about relevance** (partial relevance, studies from 6 countries: Nigeria, Malawi, Mali, Ghana, Kenya, Ethiopia) | **Moderate concerns regarding coherence** | **Moderate concerns about adequacy of data** (limited, slightly thick data from 5 studies) | **Low** | This finding was graded as Low confidence because of Moderate concerns regarding methodological limitations, coherence and adequacy of data, in addition to high concerns about relevance |
| Provider knowledge and practice | 21,24,28 | **Moderate methodological limitations (**2 studies with minor concerns, 1 study with moderate concerns) | **High concerns about relevance** (partial relevance, studies from 3 countries: Malawi, Uganda, Ethiopia) | **Minor concerns regarding coherence** | **High concerns about adequacy of data** (limited, thin data from 3 studies) | **Low** | This finding was graded as Low confidence because of Moderate concerns regarding methodological limitations and high concerns about relevance and adequacy of data |
| Lack of training on breast cancer | 24 | **Minor methodological limitations (**1 study with minor concerns) | **High concerns about relevance** (partial relevance, study from 1 country: Uganda) | **Extent of coherence unclear due to limited data** | **High concerns about adequacy** (limited, thin data from 1 study) | **Low** | This finding was graded as Low confidence because of high concerns about relevance and adequacy of data |
| Unavailability/shortage of doctors | 22,23 | **Minor methodological limitations** (both studies with minor concerns) | **High concerns about relevance** (partial relevance, studies from 2 countries: Mali, Ghana) | **Minor concerns regarding coherence** | **High concerns about adequacy** of data (limited, thin data from 2 studies) | **Moderate** | This finding was graded as Moderate confidence because of Minor concerns regarding methodological limitations and coherence, in addition to high concerns about relevance and adequacy of data |
| Workload of doctors | 23 | **Minor methodological limitations (**1 study with minor concerns) | **High concerns about relevance** (partial relevance, study from 1 country: Ghana) | **Extent of coherence unclear due to limited data** | **High concerns about adequacy** of data (limited, thin data from 1 study) | **Low** | This finding was graded as Low confidence because of high concerns about relevance and adequacy of data |
| Strikes by hospital staff | 17 | **Moderate methodological limitations** (1 study with moderate concerns) | **High concerns about relevance** (partial relevance, study from 1 country: Nigeria) | **Extent of coherence unclear due to limited data** | **High concerns about adequacy of data** (limited, thin data from 1 study) | **Low** | This finding was graded as Low confidence because of Moderate concerns regarding methodological limitations and high concerns about relevance and adequacy of data |
| **Financing** |  |  |  |  |  |  |  |
| Cost of care | 17,23,25, 26,27,28 | **Moderate methodological limitations** (4 studies with minor concerns, 2 studies with moderate concerns) | **High concerns about relevance** (partial relevance, studies from 4 countries: Nigeria, Ghana, Kenya, Ethiopia) | **Minor concerns regarding coherence** | **Moderate concerns about adequacy of data** (limited, slightly thick data from 8 studies) | **Moderate** | This finding was graded as Moderate confidence because of Moderate concerns regarding methodological limitations and adequacy of data, in addition to high concerns about relevance |
| Insurance coverage | 25,26 | **Moderate methodological limitations** (1 study with minor concerns, 1 study with moderate concerns) | **High concerns about relevance** (partial relevance, studies from 2 countries: Ghana, Kenya) | **Minor concerns regarding coherence** | **High concerns about adequacy of data** (limited, thin data from 2 studies) | **Low** | This finding was graded as Low confidence because of Moderate concerns regarding methodological limitations and high concerns about relevance and adequacy of data |
|  |  |  |  |  |  |  |  |
| **Medication Access and technologies** |  |  |  |  |  |  |  |
| Default of histologies | 17 | **Moderate methodological limitations** (1 study with moderate concerns) | **High concerns about relevance** (partial relevance, study from 1 country: Nigeria) | **Extent of coherence unclear due to limited data** | **High concerns about adequacy** of data (limited, thin data from 1 study) | **Low** | This finding was graded as Low confidence because of Moderate concerns regarding methodological limitations and high concerns about relevance and adequacy of data |
| Medical equipment failure | 21,23 | **Moderate methodological limitations (**1 study with minor concerns, 1 study with moderate concerns) | **High concerns about relevance** (partial relevance, studies from 2 countries: Malawi, Ghana) | **Extent of coherence unclear due to limited data** | **High concerns about adequacy of data** (limited, thin data from 2 studies) | **Low** | This finding was graded as Low confidence because of Moderate concerns regarding methodological limitations and high concerns about relevance and adequacy of data |
| Availability and access to medication | 21,22,23,26 | **Moderate methodological limitations** (2 studies with minor concerns, 2 studies with moderate concerns) | **High concerns about relevance** (partial relevance, studies from 4 countries: Malawi, Mali, Ghana, Kenya) | **Extent of coherence unclear due to limited data** | **High concerns about adequacy of data** (limited, thin data from 4 studies) | **Low** | This finding was graded as Low confidence because of Moderate concerns regarding methodological limitations and high concerns about relevance and adequacy of data |
|  |  |  |  |  |  |  |  |
|  |  |  |  |  |  |  |  |
| **Governance and leadership** |  |  |  |  |  |  |  |
| Low priorisation of NCDs | 24 | **Minor methodological limitations (**1 study with minor concerns) | **High concerns about relevance** (partial relevance, study from 1 country: Uganda) | **Extent of coherence unclear due to limited data** | **High concerns about adequacy** (limited, thin data from 1 study) | **Low** | This finding was graded as Low confidence because of high concerns about relevance and adequacy of data |
| Lack of cancer policy | 24,28 | **Minor methodological limitations** (both studies with minor concerns) | **High concerns about relevance** (partial relevance, studies from 2 countries: Uganda, Ethiopia) | **Minor concerns regarding coherence** | **High concerns about adequacy** (limited, thin data from 2 studies) | **Moderate** | This finding was graded as Moderate confidence because of Minor concerns regarding methodological limitations and coherence, in addition to high concerns about relevance and adequacy of data |
| Lack of cancer services | 24,27,28 | **Minor methodological limitations** (the 3 studies with minor concerns) | **High concerns about relevance** (partial relevance, studies from 2 countries: Uganda, Ethiopia) | **Extent of coherence unclear due to limited data** | **High concerns about adequacy** (limited, thin data from 3 studies) | **Low** | This finding was graded as Low confidence because of high concerns about relevance and adequacy of data |

**Table S4: Search strategy (from April to 26^th^ July 2020)**

| **Data sources** | **Search Terms/Equations** |
| --- | --- |
| PubMed (Filters :10 years; Humans) | (((“breast cancer” [Mesh] OR “breast carcinoma” "[Mesh] OR “breast neoplasm” "[Mesh] OR “breast tumor” "[Mesh]) AND (factors [All Fields]OR determinants [All Fields]OR barriers [All Fields] OR challenges OR facilitators OR opportunities)) AND (delayed treatment OR time-to-treatment[Mesh] OR provider delay OR doctor delay OR treatment delay OR healthcare delivery OR healthcare access OR health system OR health service accessibility)) AND (Africa OR Sub-Saharan Africa OR South Africa OR Angola OR Botswana OR Burkina Faso OR Burundi OR Benin OR Cabo Verde OR Cameroon OR Comoros OR Republic of Congo OR Democratic Republic of Congo OR Ivory Cost OR Eswatini OR Gabon OR Gambia OR Ghana OR Guinea OR Equatorial Guinea OR Guinea-Bissau OR Kenya OR Lesotho OR Liberia OR Madagascar OR Malawi OR Mali OR Mauritius OR Mauritania OR Mozambique OR Namibia OR Niger OR Nigeria OR Uganda OR Rwanda OR Central African Republic OR Sao Tome OR Principe OR Seychelles OR Sierra Leone OR Somalia OR Sudan OR South Sudan OR Senegal OR Tanzania OR Chad OR Togo OR Zambia OR Zimbabwe OR Eritrea OR Ethiopia) |
|  | "breast cancer"[Mesh] AND determinants AND time-to-treatment [Mesh] AND Sub-Saharan Africa [Mesh] |
|  | "breast carcinoma" AND determinants AND time-to-treatment AND Sub-Saharan Africa |
|  | "breast tumor" AND determinants AND time-to-treatment AND Sub-Saharan Africa |
|  | "breast neoplasm" AND determinants AND time-to-treatment AND Sub-Saharan Africa |
|  | "breast cancer" AND factors AND treatment delay AND Sub-Saharan Africa |
|  | "breast cancer" AND factors AND delayed treatment AND Sub-Saharan Africa |
|  | "breast cancer" AND factors AND provider delay AND Sub-Saharan Africa |
|  | "breast cancer" AND factors AND doctor delay AND Sub-Saharan Africa |
|  | "breast cancer" AND barriers AND healthcare delivery AND Sub-Saharan Africa |
|  | "breast cancer" AND barriers AND healthcare access AND Sub-Saharan Africa |
|  | "breast cancer" AND barriers AND health system AND Sub-Saharan Africa |
|  | "breast cancer" AND barriers AND health service accessibility AND Sub-Saharan Africa |
|  | "breast cancer" AND challenges AND health service accessibility AND Sub-Saharan Africa |
|  | "breast cancer" AND facilitators AND health service accessibility AND Sub-Saharan Africa |
|  | "breast cancer" AND opportunities AND health service accessibility AND Sub-Saharan Africa |
|  | "breast cancer" AND challenges AND healthcare access AND Sub-Saharan Africa |
|  | "breast cancer" AND challenges AND healthcare delivery AND Sub-Saharan Africa |
|  | "breast cancer" AND challenges AND health system AND Sub-Saharan Africa |
|  | "breast cancer" AND facilitators AND health system AND Sub-Saharan Africa |
|  | "breast cancer" AND opportunities AND health system AND Sub-Saharan Africa |
|  | "breast cancer" AND opportunities AND healthcare access AND Sub-Saharan Africa |
|  | "breast cancer" AND opportunities AND healthcare delivery AND Sub-Saharan Africa |
|  | "breast cancer" AND facilitators AND healthcare delivery AND Sub-Saharan Africa |
|  | "breast cancer" AND facilitators AND healthcare access AND Sub-Saharan Africa |
|  | "breast cancer" AND determinants AND treatment delay AND Sub-Saharan Africa |
|  | "breast cancer" AND determinants AND delayed treatment AND Sub-Saharan Africa |
|  | "breast cancer" AND determinants AND doctor delay AND Sub-Saharan Africa |
|  | "breast cancer" AND determinants AND provider delay AND Sub-Saharan Africa |
|  | "breast cancer" AND determinants AND healthcare delivery AND Sub-Saharan Africa |
|  | "breast cancer" AND determinants AND healthcare access AND Sub-Saharan Africa |
|  | "breast cancer" AND determinants AND health system AND Sub-Saharan Africa |
|  | "breast cancer" AND determinants AND health service accessibility AND Sub-Saharan Africa |
|  | "breast cancer" AND factors AND health system AND Sub-Saharan Africa |
|  | "breast cancer" AND factors AND healthcare delivery AND Sub-Saharan Africa |
|  | "breast cancer" AND factors AND healthcare access AND Sub-Saharan Africa |
|  | "breast cancer" AND factors AND health service accessibility AND Sub-Saharan Africa |
|  | "breast cancer" AND factors AND time-to-treatment AND Sub-Saharan Africa |
|  | breast cancer AND barriers AND treatment delay AND Africa |
|  | breast cancer AND opportunities AND treatment delay AND Africa |
|  | breast cancer AND factors AND time to treatment AND Africa |
|  | facilitators AND health system delay AND breast cancer AND Africa |
|  | health service accessibility AND breast cancer AND Africa |
|  | determinants AND delayed treatment AND breast cancer AND Africa |
|  | factors AND delayed treatment AND breast cancer AND Africa |
|  | ((("Breast Neoplasms"[Mesh]) AND "Time-to-Treatment"[Mesh]) AND "Delivery of Health Care"[Mesh]) AND "Africa"[Mesh] |
|  | barriers AND breast cancer AND healthcare access AND Africa |
|  | breast tumor AND healthcare access AND Africa |
|  | doctor delay AND determinants AND Breast tumor AND healthcare access AND Africa |
|  | doctor delay AND factors AND breast cancer AND healthcare access AND Africa |
|  | provider delay AND healthcare access AND breast tumor AND Africa |
|  | challenges AND breast cancer AND healthcare access AND Africa |
|  | breast cancer AND delayed treatment AND Africa |
| Mendeley (Date, articles, journal, generic) | breast cancer AND treatment delay AND Africa |
|  | breast cancer AND Sub-Saharan Africa |
|  | breast cancer AND time-to-treatment AND Sub-Saharan Africa |
|  | breast cancer AND treatment delay AND Sub-Saharan Africa |
| Google Scholar (2010-2020) | breast cancer AND time-to-treatment AND Sub-Saharan Africa |
|  | "breast cancer" AND (determinants OR challenges OR opportunities) AND (treatment delay OR time-to-treatment) AND (Sub-Saharan Africa) NOT male NOT genes NOT receptors |
| Science direct (2010-2020; Research articles) | breast cancer AND treatment delay AND Africa |
|  | “breast cancer” AND delayed treatment AND Sub-Saharan Africa |
| AJOL (African Journal On Line) | breast cancer AND treatment delay AND Africa |
|  | breast cancer AND Sub-Saharan Africa |
| ResearchGate | breast cancer AND Sub-Saharan Africa |
|  | breast cancer AND time-to-treatment AND Sub-Saharan Africa |
|  | breast cancer AND treatment delay AND Sub-Saharan Africa |
|  | breast cancer AND challenges AND Sub-Saharan Africa |
|  | breast cancer AND barriers AND Sub-Saharan Africa |
|  | Country-by-country search ( 48 countries): breast cancer AND Country (South Africa; Angola; Botswana; Burkina Faso; Burundi; Benin; Cabo Verde; Cameroon; Comoros; Republic of Congo; Democratic Republic of Congo; Ivory Cost; Eswatini; Gabon; Gambia; Ghana; Guinea; Equatorial Guinea; Guinea-Bissau; Kenya; Lesotho; Liberia; Madagascar; Malawi; Mali; Mauritius; Mauritania; Mozambique; Namibia; Niger; Nigeria; Uganda; Rwanda; Central African Republic; Sao Tome; Principe; Seychelles; Sierra Leone; Somalia; Sudan; South Sudan; Senegal; Tanzania; Chad; Togo; Zambia; Zimbabwe; Eritrea; Ethiopia) |

**Table S5: List of full-text articles excluded and reasons for exclusion (n = 39)**

| **List of full-text articles excluded** | **Reasons for exclusion** |
| --- | --- |
| **Schleimer et al (2019)** [1] | Study including male breast cancer |
| **Price et al (2012)** [2] | Study including male breast cancer |
| **Pace et al (2019)** [3] | Study including male breast cancer |
| **Otieno et al (2010)** [4] | Study including male breast cancer |
| **Ezeome (2010)** [5] | Study including male breast cancer |
| **Dedey et al (2016)** [6] | Study including male breast cancer |
| **Anakwenze et al (2017)** [7] | Not specific to breast cancer |
| **Long et al (2015)** [8] | Not specific to breast cancer |
| **Grosse Frie et al (2019)** [9] | Not specific to breast cancer |
| **Leng et al (2020)** [10] | Not specific to breast cancer |
| **Ntirenganya et al (2014)** [11] | Not specific to breast cancer |
| **Tapela et al (2016)** [12] | Not specific to breast cancer |
| **Haileselassie et al (2019)** [13] | Not specific to breast cancer |
| **Mandizadza et al (2015)** [14] | Not specific to breast cancer |
| **Anyanwu et al ( 2011)** [15] | Don’t answer our research question |
| **Ale et al (2019)** [16] | Don’t answer our research question |
| **Feuchtner et al (2019)** [17] | Don’t answer our research question |
| **Deressa et al (2019)** [18] | Don’t answer our research question |
| **Hjelm et al (2019)** [19] | Don’t answer our research question |
| **O'Neil et al (2019)** [20] | Don’t answer our research question |
| **Tesfamariam et al (2013)** [21] | Don’t answer our research question |
| **Ersumo et al (2018)** [22] | Don’t answer our research question |
| **Umoke et al (2019)** [23] | Don’t answer our research question |
| **Akinkuolie et al (2016)** [24] | Don’t answer our research question |
| **Mutebi et al (2017)** [25] | Don’t answer our research question |
| **Mubiligi et al (2014)** [26] | Don’t answer our research question |
| **Dye et al (2010)** [27] | Don’t answer our research question |
| **Egwuonwu et al (2012)** [28] | Don’t answer our research question |
| **Agodirin et al (2017)** [29] | Don’t answer our research question |
| **Rayne et al (2019)** [30] | Don’t answer our research question |
| **Brinton et al (2017)** [31] | Don’t answer our research question |
| **Takongmo et al (2011)** [32] | Don’t answer our research question |
| **O'Neil et al (2019)** [33] | Don’t answer our research question |
| **Rick et al (2019)** [34] | Don’t answer our research question |
| **Dusengimana et al (2018)** [35] | Don’t answer our research question |
| **Otieno et al (2010)** [36] | Don’t answer our research question |
| **Dalwai et al (2015)** [37] | Don’t answer our research question |
| **Chalya et al (2014)** [38] | Don’t answer our research question |
| **Rayne et al (2019)** [39] | Don’t answer our research question |

**References for Supplementary Material**

1. Schleimer L, Dusengimana J-M, Butonzi J, Kigonya C, Natarajan A, Umwizerwa A, et al. Barriers to timely surgery for breast cancer in Rwanda. Surgery. 2019;166.

2. Price AJ, Ndom P, Atenguena E, Mambou Nouemssi JP, Ryder RW. Cancer care challenges in developing countries: Cancer Challenges in Cameroon. Cancer. 2012;118:3627–35. doi:10.1002/cncr.26681.

3. Pace LE, Dusengimana JMV, Shulman LN, Schleimer LE, Shyirambere C, Rusangwa C, et al. Cluster Randomized Trial to Facilitate Breast Cancer Early Diagnosis in a Rural District of Rwanda. Journal of global oncology. 2019;5:1–13.

4. Otieno ES, Micheni JN, Kimende SK, Mutai KK. Delayed presentation of breast cancer patients. East African Medical Journal. 2010;87:147–50. doi:10.4314/eamj.v87i4.62410.

5. Emmanuel R Ezeome. Delays in presentation and treatment of breast cancer in Enugu, Nigeria. ResearchGate. https://www.researchgate.net/publication/46392202_Delays_in_presentation_and_treatment_of_breast_cancer_in_Enugu_Nigeria.

6. Dedey et al. Factors Associated With Waiting Time for Breast Cancer Treatment in a Teaching Hospital in Ghana. ResearchGate. 2020. https://www.researchgate.net/publication/301483829_Factors_Associated_With_Waiting_Time_for_Breast_Cancer_Treatment_in_a_Teaching_Hospital_in_Ghana.

7. Anakwenze CP, Ntekim A, Trock B, Uwadiae IB, Page BR. Barriers to radiotherapy access at the University College Hospital in Ibadan, Nigeria. Clinical and translational radiation oncology. 2017;5:1–5.

8. Long C, Titus Ngwa Tagang E, Popat RA, Lawong EK, Brown JA, Wren SM. Factors associated with delays to surgical presentation in North-West Cameroon. Surgery. 2015;158:756–63. doi:10.1016/j.surg.2015.04.016.

9. Grosse Frie et al. Health system organisation and patient pathways: breast care patients’ trajectories and medical doctors’ practice in Mali. 2019 BMC Public Health 19.10.1186/s12889-019-6532-8. ResearchGate. https://www.researchgate.net/publication/331186396_Health_system_organisation_and_patient_pathways_breast_care_patients%27_trajectories_and_medical_doctors%27_practice_in_Mali.

10. Leng J, Ntekim AI, Ibraheem A, Anakwenze CP, Golden DW, Olopade OI. Infrastructural Challenges Lead to Delay of Curative Radiotherapy in Nigeria. JCO Glob Oncol. 2020;6. doi:10.1200/JGO.19.00286.

11. Ntirenganya F, Petroze RT, Kamara TB, Groen RS, Kushner AL, Kyamanywa P, et al. Prevalence of breast masses and barriers to care: results from a population-based survey in Rwanda and Sierra Leone. J Surg Oncol. 2014;110:903–6.

12. Tapela NM, Mpunga T, Hedt-Gauthier B, Moore M, Mpanumusingo E, Xu MJ, et al. Pursuing equity in cancer care: implementation, challenges and preliminary findings of a public cancer referral center in rural Rwanda. BMC Cancer. 2016;16:237.

13. Haileselassie W, Mulugeta T, Tigeneh W, Kaba M, Labisso WL. The situation of cancer treatment in Ethiopia: challenges and opportunities. Journal of cancer prevention. 2019;24:33.

14. Mandizadza EJ, Rusakaniko S. Understanding delayed presentation among people diagnosed with cancer in Zimbabwe: a phenomenological view. Journal of Social Development in Africa. 2015;30:71–94.

15. Anyanwu SNC, Egwuonwu OA, Ihekwoaba EC. Acceptance and adherence to treatment among breast cancer patients in Eastern Nigeria. Breast. 2011;20 Suppl 2:S51-53.

16. Ale AF, Isichei MW, Misauno MA. Access to Breast Cancer Care in Jos, North Central. Journal of Advances in Medicine and Medical Research. 2019;:1–7. doi:10.9734/jammr/2019/v31i830317.

17. Feuchtner J, Mathewos A, Solomon A, Timotewos G, Aynalem A, Wondemagegnehu T, et al. Addis Ababa population-based pattern of cancer therapy, Ethiopia. PLoS One. 2019;14. doi:10.1371/journal.pone.0219519.

18. Deressa BT, Cihoric N, Badra EV, Tsikkinis A, Rauch D. Breast cancer care in northern Ethiopia - cross-sectional analysis. BMC Cancer. 2019;19:393.

19. Hjelm TE, Matovu A, Mugisha N, Löfgren J. Breast cancer care in Uganda: A multicenter study on the frequency of breast cancer surgery in relation to the incidence of breast cancer. ResearchGate. 2019. https://www.researchgate.net/publication/334404891_Breast_cancer_care_in_Uganda_A_multicenter_study_on_the_frequency_of_breast_cancer_surgery_in_relation_to_the_incidence_of_breast_cancer.

20. O’Neil D et al. Breast Cancer Care Quality in South Africa’s Public Health System: An Evaluation Using American Society of Clinical Oncology/National Quality Forum Measures. ResearchGate. 2019. https://www.researchgate.net/publication/337553582_Breast_Cancer_Care_Quality_in_South_Africa%27s_Public_Health_System_An_Evaluation_Using_American_Society_of_Clinical_OncologyNational_Quality_Forum_Measures.

21. Tesfamariam A, Gebremichael A, Mufunda J. Breast cancer clinicopathological presentation, gravity and challenges in Eritrea, East Africa: Management practice in a resource-poor setting. ResearchGate. 2013. doi:http://dx.doi.org/10.7196/samj.6829.

22. Ersumo T et al. Breast cancer in a private medical services center: A 10-year experience. 2018. https://www.mendeley.com/catalogue/7a650f46-6490-3de8-a514-f17a420adb24/.

23. Umoke IC, Garba ES. Breast cancer in North-Central Nigeria: challenges to good management outcome. ResearchGate. 2019. https://www.researchgate.net/publication/335460245_Breast_cancer_in_North-Central_Nigeria_challenges_to_good_management_outcome.

24. Akinkuolie AA, Etonyeaku AC, Olasehinde O, Arowolo OA, Babalola RN. Breast cancer patients’ presentation for oncological treatment: a single centre study. Pan Afr Med J. 2016;24:63.

25. Mutebi M, Simonds H. Breast ductal carcinoma in situ in an unscreened population: presentation, diagnosis and management at a single tertiary centre. S Afr J Surg. 2017;55:4–9.

26. Mubiligi J, al. Caring for patients with surgically resectable cancers: experience from a specialised centre in rural Rwanda. ResearchGate. 2014. https://www.researchgate.net/publication/272144377_Caring_for_patients_with_surgically_resectable_cancers_experience_from_a_specialised_centre_in_rural_Rwanda.

27. Dye TD, Bogale S, Hobden C, Tilahun Y, Hechter V, Deressa T, et al. Complex care systems in developing countries: breast cancer patient navigation in Ethiopia. Cancer: Interdisciplinary International Journal of the American Cancer Society. 2010;116:577–585.

28. Egwuonwu OA, Anyanwu SNC, Nwofor AME. Default from neoadjuvant chemotherapy in premenopausal female breast cancer patients: what is to blame? Niger J Clin Pract. 2012;15:265–9.

29. Agodirin O. Delay between Breast Cancer Detection and Arrival at Specialist Clinic Preliminary Revelations of Multicentered Survey in Nigeria. TEXILA INTERNATIONAL JOURNAL OF PUBLIC HEALTH. 2017;5:552–7.

30. Rayne S, Schnippel K, Kruger D, Benn C, Firnhaber C. Delay to diagnosis and breast cancer stage in an urban South African breast clinic. South African Medical Journal. 2019;109:159.

31. Brinton L, Figueroa J, Adjei E, Ansong D, Biritwum R, Edusei L, et al. Factors contributing to delays in diagnosis of breast cancers in Ghana, West Africa. Breast cancer research and treatment. 2017;162:105–114.

32. Takongmo S et al. Neoadjuvant chemotherapy in the treatment of advanced and inflammatory breast cancer in Yaoundé (Cameroon). 2011. https://www.mendeley.com/catalogue/7fea319f-13da-3175-9f85-55f82708fc4d/.

33. O’Neil DS, Nietz S, Buccimazza I, Singh U, Čačala S, Stopforth LW, et al. Neoadjuvant chemotherapy use for nonmetastatic breast cancer at five public South African Hospitals and impact on time to initial cancer therapy. The oncologist. 2019;24:933.

34. Rick T, Habtamu B, Tigeneh W, Abreha A, van Norden Y, Grover S, et al. Patterns of Care of Cancers and Radiotherapy in Ethiopia. J Glob Oncol. 2019;5:1–8.

35. Dusengimana JMV, Hategekimana V, Borg R, Hedt-Gauthier B, Gupta N, Troyan S, et al. Pregnancy-associated breast cancer in rural Rwanda: the experience of the Butaro Cancer Center of Excellence. BMC Cancer. 2018;18:1–8. doi:10.1186/s12885-018-4535-y.

36. Otieno ES, Micheni JN, Kimende SK, Mutai KK. Provider delay in the diagnosis and initiation of definitive treatment for breast cancer patients. East African Medical Journal. 2010;87:143–6. doi:10.4314/eamj.v87i4.62201.

37. Dalwai E, Buccimazza I. System delays in breast cancer. ResearchGate. 2015. https://www.researchgate.net/publication/282588947_System_delays_in_breast_cancer.

38. Chalya P, Lema M, Mabula J, Rambau P, Mchembe M, Masalu N, et al. Triple assessment as a preoperative diagnostic tool for breast cancer at Bugando Medical Centre in northwestern Tanzania. Tanzania Journal of Health Research. 2014;15.

39. Rayne S, Schnippel K, MD S, Fearnhead K, Kruger D, Benn C, et al. Unraveling the South African Breast Cancer Story: The Relationship of Patients, Delay to Diagnosis, and Tumor Biology With Stage at Presentation in an Urban Setting. Journal of Surgical Research. 2019;235:181–9.
